# Pathological Complete Remission of Pancreatic Cancer Following Neoadjuvant Chemoradiation Therapy; Not the End of Battles

**DOI:** 10.1097/MD.0000000000002168

**Published:** 2015-12-31

**Authors:** Sung Hwan Lee, Chang Moo Kang, Hogeun Kim, Ho Kyoung Hwang, Si Young Song, Jinsil Seong, Myoung Jin Kim, Woo Jung Lee

**Affiliations:** From the Department of Surgery, Yonsei University College of Medicine, Seoul, South Korea (SHL, CMK, HKH, WJL); Department of Pathology, Yonsei University College of Medicine, Seoul, South Korea (HK); Department of Gastroenterology, Yonsei University College of Medicine, Seoul, South Korea (SYS); Department of Radiation Oncology, Yonsei University College of Medicine, Seoul, South Korea (JSS); Department of Radiology, Yonsei University College of Medicine, Seoul, South Korea (MJK).

## Abstract

In spite of controversial issues, pancreatectomy following neoadjuvant chemoradiation therapy (NeoCRT) has been applied in treating advanced pancreatic cancer. Cases of pathological complete remission (pCR) following NeoCRT is rare, and its long-term follow-up data are still lacking.

From January 2000 to December 2012, medical records of the patients who underwent pancreatectomy for pancreatic ductal adenocarcinoma were retrospectively reviewed. Characteristics of the patients with pCR were summarized and their long-term follow-up data were analyzed.

Among 86 patients with pancreatic cancer who underwent radical pancreatectomy following NeoCRT, 10 patients (11.6%) were reported to pCR. Nine out of 10 patients received gemcitabine-based chemoradiation therapy. Median pre-NeoCRT serum CA 19-9 was 313.5 U/ml, and post-NeoCRT serum CA 19-9 was 9.9 U/ml, which was shown to be significant difference between 2 serum CA 19-9 level (*P* = 0.005). Pylorus-preserving pancreaticoduodenectomy was done in 8 patients, and the others received distal pancreatosplenectomy. Postoperative chemotherapy was received in 6 patients. Disease-free survival was statistically superior in patients with pCR than patients without pCR (*P* < 0.05). However, 5 patients experienced cancer recurrence and no clinicopathologic variables including preoperative resectability could not predict the potential recurrence of tumor in patients with pCR (*P* > 0.05).

pCR is rarely reported following NeoCRT, but this condition is not telling the cure of the disease. Early recurrence in the pattern of liver metastasis and peritoneal seeding can be expected. However, long-term survival could be maintained in patients without recurrence. Further investigation is necessary for predicting failure of treatment.

## INTRODUCTION

Pancreatic cancer is regarded as one of the lethal malignant diseases arising in gastrointestinal tract. Margin-negative resection is an essential step for cure of disease, but only 20% of the patients with pancreatic cancer can be treated by surgical resection.^1^ However, most patients who underwent pancreatectomy usually experience tumor recurrence, especially in liver, within 2 years after surgery, resulting in disease-specific survival, 15% to 20%.^2^ Therefore, surgery alone is not enough for cure of pancreatic cancer. Postoperative adjuvant chemotherapy should be applied for improving oncologic outcomes. Unfortunately, it is reported as many as 50% of the patients who underwent curative resection of pancreatic cancer cannot receive proper postoperative adjuvant chemotherapy due to delayed recovery and major surgical morbidity.^3^

There are several rationales of neoadjuvant therapy (Neo-Tx) in pancreatic cancer. It can convert an initial unresectable pancreatic cancer to a resectable one. It can avoid unnecessary laparotomy if the pancreatic cancer progresses during preoperative treatment. It provides complete cancer treatment in case of surgical resection. The effect of preoperative anticancer treatment can be enhanced because tissues are well vascularized. Recent large volume series suggest that pancreatectomy following Neo-Tx is safe and effective in treating advanced pancreatic cancer.^4,5^ Oncologic role of pancreatectomy following Neo-Tx in treating pancreatic cancer is sum of following effects: patients selection, potential down-staging effect, and complete cancer treatment.^6^

Pathologic complete remission (pCR) after Neo-Tx has been reported in treating other gastrointestinal cancer. In rectal cancer patients who have received Neo-Tx, approximately 15% of the patients are reported to be pCR,^7^ and patients with esophageal cancer who received Neo-Tx, up to 40% of them have been reported to have pCR in their surgical specimens. It was shown that pCR improved oncologic outcomes including lower incidence of local recurrence, distant metastasis, and better survival rate.^8^

However, there are a few case reports on pCR following Neo-Tx in treating pancreatic cancer.^9^ According to literatures, about 1.3% to 7% of the patients with pancreatectomy following Neo-Tx are reported to be complete remission (pT0).^4,5^ The prognostic value of pCR in pancreatic cancer is not still clearly identified. In this study, we identified 9 patients with pCR among 86 patients who underwent pancreatectomy following Neo-Tx for pancreatic cancer. We investigated prognostic impact of pCR on oncologic outcome of the pancreatic cancer.

## MATERIALS AND METHODS

### Patients and Data Collection

This study was approved by the Institutional Review Board of Severance hospital. From January 2000 to December 2012, we retrospectively evaluated the patients who underwent surgical resection following neoadjuvant treatment for pancreatic cancer from our cohort database. Demographic and clinicopathologic factors were acquired from database and the information for disease-free and disease-specific overall survival period were also included.

### Neoadjuvant Treatment for Pancreatic Cancer

Neoadjuvant treatments were applied for borderline resectable pancreatic cancer in most cases. After the pathologic confirmation regarding pancreatic ductal adenocarcinoma using cytology via endoscopic retrograde cholangiography, patients received gemcitabine-based chemotherapy including or not additional radiotherapy of which total radiation dose was usually from 5040 to 6000 cGy. Pancreatectomy was performed after evaluation for treatment effect in radiologic evaluations.

### Statistics

All statistical analyses were performed by using IBM® SPSS® Statistics version 20. Continuous variables were indicated as mean ± standard deviation or range and categorical variables as frequency and percentage. Student *t* test and Mann–Whitney U test were used for comparing continuous variables between pCR and non-pCR group and Chi-square and Fisher exact were used for comparing categorical data between 2 groups. Kaplan–Meier method was applied for survival analysis of disease-free and disease-specific overall survival period. *P*-value < 0.05 was considered as statistical significance.

## RESULTS

### Patients Demographics of PCR Confirmed by Pancreatectomy Following Neo-CRT

**FIGURE 1:**
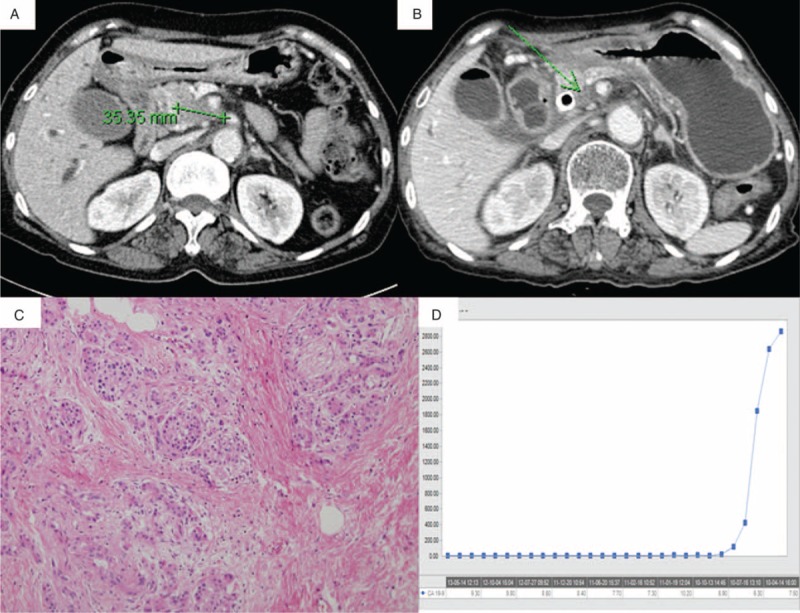
Clinical feature of pathologic complete remission of pancreatic cancer after NeoCRT. (A) Pancreatic cancer in uncinate portion with SMA abutment before Neo-Tx, (B) shrunk uncinate mass after Neo-Tx, (C) no residual cancer cell in pathologic examination, (D) a steep decline of CA 19-9 level after Neo-Tx. ∗NeoCRT, neoadjuvant chemoradiation therapy; SMA, superior mesenteric artery.

Among 86 patients who underwent pancreatectomy following Neo-CRT, 9 patients (10.4%) were reported to have no residual pancreatic cancer cells in final pathologic examination (Figure 1, Table 1). Six patients were female and 3 were male with median age, 64 (range, 55–74) years. Most patients (8 out of 9 patients) received gemcitabine-based chemotherapy or chemoradiation therapy. Initial CA 19-9 was noted to be 685.1 ± 1024.7, which was decreased to 20.1 ± 30.1 following neoadjuvant chemoradiation therapy (NeoCRT) (*P* = 0.008). Ten patients had pancreatic head cancer requiring pancreaticoduodenectomy. Five patients required combined venous vascular resection such as 4 segmental resections of SMV-SV-PV confluence and 1 wedge resection of SMV.

**TABLE 1:**
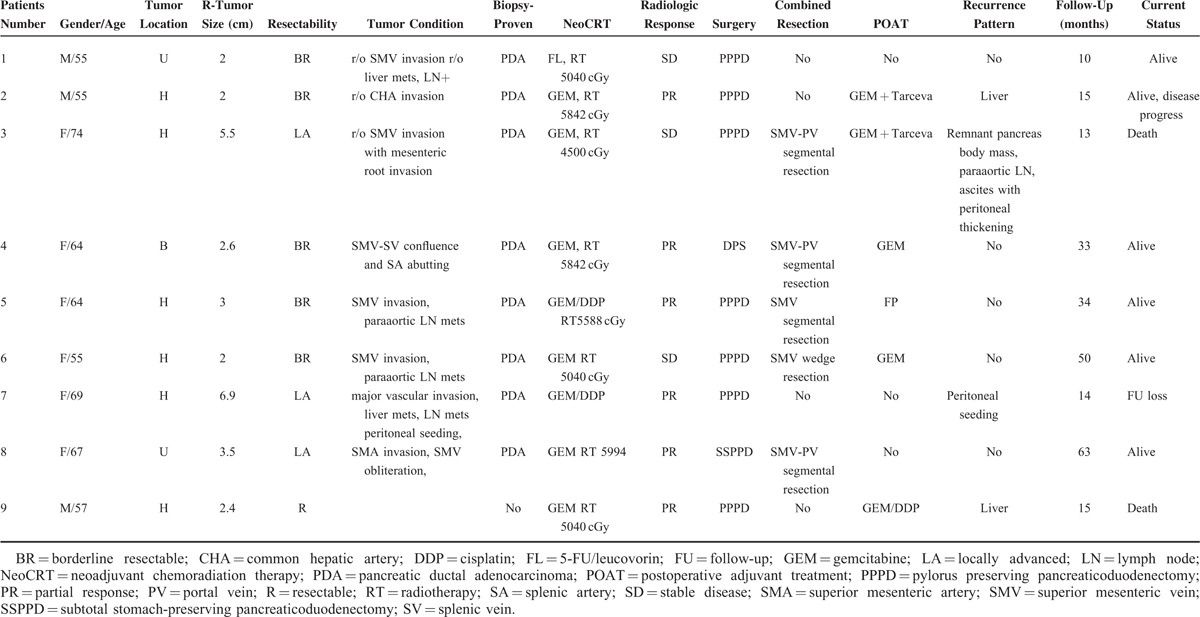
Case Series of pCR Following Neo-CRT

### Comparison of Clinicopathologic Factors Between pCR and Non-pCR

**FIGURE 2:**
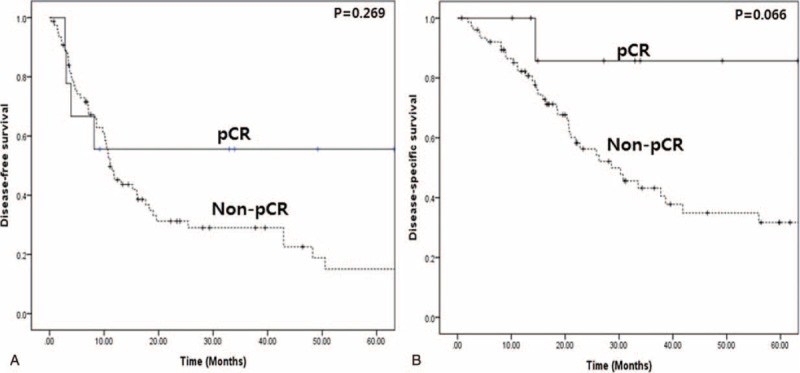
Oncologic outcome of pancreatectomy following Neo-CRT. (A) Comparisons of disease-free survival of pCR and non-pCR groups by Kaplan–Meier method (*P* = 0.269), (B) comparisons of disease-specific overall survival of pCR and non-pCR groups (*P* = 0.066). ∗pCR, pathological complete remission.

In comparative analysis, there were no clinicopathologic differences between pCR and non-pCR group (*P* > 0.05), however, CA19-9 after Neo-CRT (*P* = 0.053), pT stage (*P* < 0.001), and pN stage (*P* = 0.024) were significantly different between 2 groups (Table 2). During follow-up period (median 21 months (range, 10–63)), 4 patients experienced tumor recurrence (2 in liver and 2 in peritoneum), and the rest 5 patients (5.8%, 5 out of 86) still have no evidence of tumor recurrence in pCR group. Most recurrence was found within 1 year after surgery. Among them, 4 patients have been in disease-free condition for more than approximately 3 years. There was no difference in disease-free survival (mean 37.1 months vs. 25.3 months, *P* = 0.269) between 2 groups (Figure 2A). Disease-specific survival in pCR group was shown to be superior to that of non-pCR group with marginal significance (mean, 56.3 months vs. 41.9 months, *P* = 0.066), Figure 2B.

**TABLE 2:**
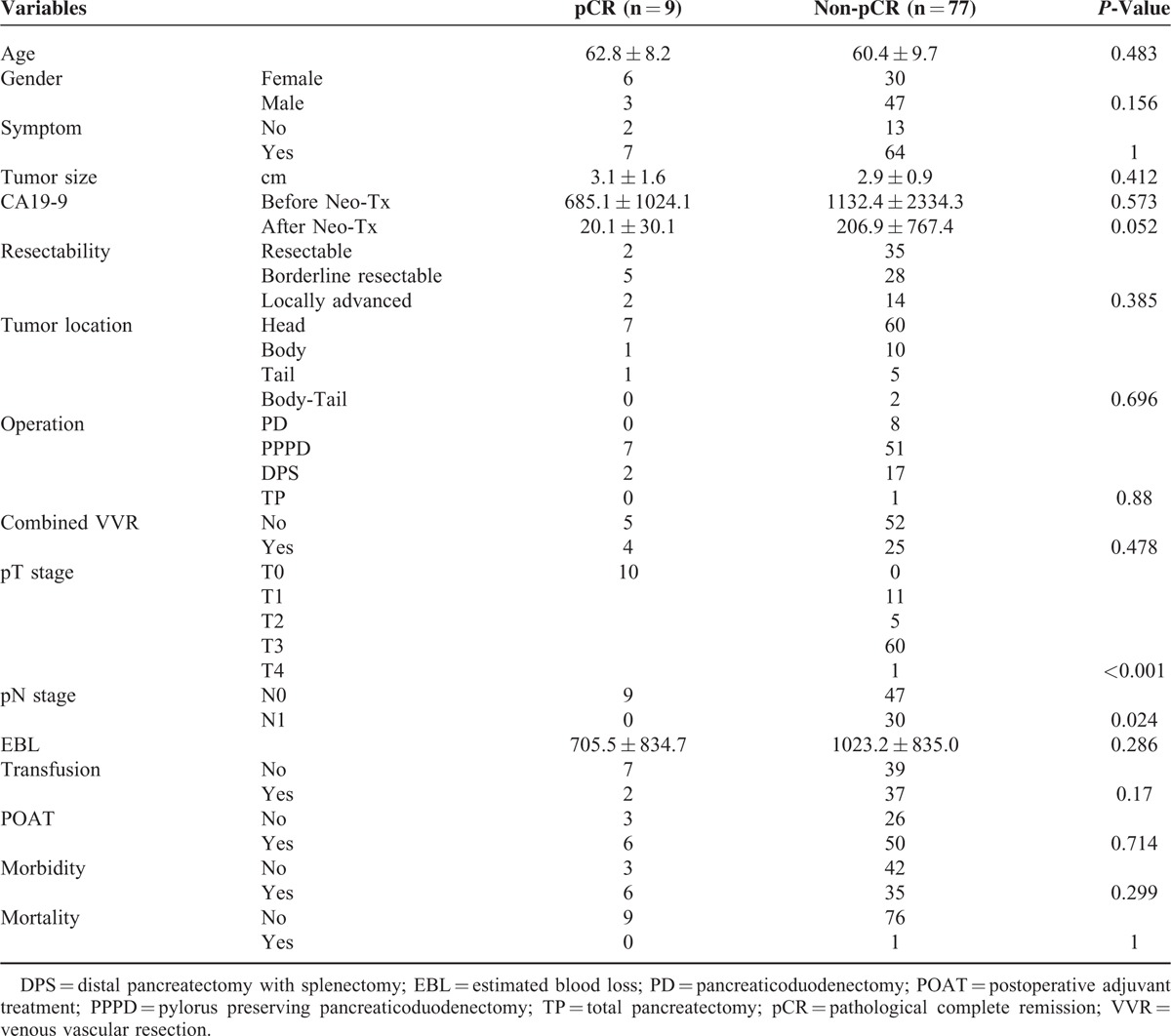
Comparison of Clinicopathologic Factors Between pCR and Non-pCR

## DISCUSSION

The current results showed that even 4 patients with pCR showed systemic recurrence in liver and peritoneum within 1 year, leading to skeptical opinion about pCR; *is pCR real?* One possible reason may be resulted from pathological underestimation or inaccurate assessment of treatment effect of Neo-Tx. There are proposed several grading systems to assess the treatment effect of Neo-Tx in pancreatic cancer by Ishikawa et al,^10^ Evans et al,^11^ Pendurthi et al,^12^ and White et al.^13^ Essentially, these grading schemes are dealing with one of the 3 variables: viable tumor cell mass, fibrosis, and/or necrosis, but providing inconsistency and controversy in determining treatment effect of Neo-Tx. For example, according to the original descriptions of tumor response from Evans scheme,^11^ pCR may represent nonviable tumor cells as grade IV response. Nonviable tumor cells were characterized as “cells with bizarre, hyperchromatic, or pyknotic nuclei, usually associated with markedly swollen, vacuolated or deeply eosinophilic cytoplasm.”^10^ It is extremely difficult to determine the viability of residual carcinoma cells based on morphologic features in clinical practice.^14^ Routine hematoxylin and eosin staining-based pathological examination may not detect potential residual cancer cell in resected specimen. In addition, there is still no world widely accepted standardized protocol in handling pancreatectomized specimen of pancreatic cancer, and the protocols for pathological examination in pancreatectomized specimen seem to vary from institutions to institutions. It may depend on individual pathologists’ insight and effort to accurately assess treatment effect of Neo-Tx in pancreatic cancer.

Therefore, there is potential room for not detecting residual cancer cells in routine pathological examinations, which later resulting in treatment failure even in cases of pCR. In fact, considering relative low incidence of pCR^4,5^ in reported literatures, 10.5% incidence of pCR looks high in present study (9 out of 86 patients).

Another potential mechanism of tumor recurrence after confirming pCR can be explained as follow; *pancreatic cancer may be systemic disease from the beginning*. Although pancreatic cancer cells in resected specimen were all destroyed due to Neo-Tx, metastatic foci refractory to conventional treatment might still exist and cause subsequent recurrence during the follow-up period. As shown in present data, 2 recurrences were found as hepatic metastasis and the rest two as peritoneal seeding, supporting this postulation.

Therefore, even patients with pCR who underwent radical pancreatectomy following Neo-Tx need to receive postoperative adjuvant chemotherapy. Considering viable metastatic tumor cells resistant to previous Neo-Tx, additional new combination of chemotherapeutic agent would be ideal in this setting, which will be further investigated in clinical oncology.

Most recurrence was found within 1 year after surgery, however, it should be noted that patients with pCR who did not experience tumor recurrence remains in disease-free condition. Five patients (5.8%, 5 out of 86) still have no evidence of tumor recurrence in pCR group. Among them, 4 patients have been in disease-free condition for more than approximately 3 years. Aside from potential contamination of undetected residual cancer cells and systemic nature of disease in pCR group, this observation is strongly suggesting *pCR, it is real* in clinical setting of Neo-Tx for pancreatic cancer. Zhao et al^15^ recently reported that 100% of disease-specific overall survival in patients with pCR (n = 10), which showing a significant better than those of posttreatment stage I or stage IIA disease in resected pancreatic cancer following Neo-Tx (*P* < 0.001). Their reported incidence of pCR is 2.5% indirectly shows strict and meticulous evaluation of pathologic examination of resected specimen. In present study, it was observed that disease-specific survival in pCR group was shown to be superior to that of non-pCR group with marginal significance (mean, 56.3 months vs. 41.9 months, *P* = 0.066), but when considering pure pCR candidates, there are significant survival difference between 2 groups (*P* < 0.05, data not shown).

Retrospective study design and small number of patients with pCR are inevitable limitations in our study. And, study period was relatively long and a number of pathologists were involved in pathologic examination for pancreatectomy specimen. Nevertheless, this study can be valuable evidence for evaluating clinical meaning of pCR following neoadjuvant treatment in pancreatic cancer in limited clinical references.

In conclusion, pCR can be achieved from neoadjuvant treatment in pancreatic cancer. Postoperative adjuvant treatment, however, may be required even in pCR because pCR can be contaminated with potential residual cancer cells and pancreatic cancer is systemic disease which can have metastatic foci in early phase. Further investigation for predicting failure of neoadjuvant treatment is mandatory.
